# Comparative analysis of adding cotton straw and corn stover to improve the combustion performance of municipal sludge

**DOI:** 10.1038/s41598-024-56842-4

**Published:** 2024-03-15

**Authors:** Feng Xu, Jing Li, Zihan He

**Affiliations:** 1 School of Civil Engineering and Architecture, Xinjiang University, Urumqu, 830049 China; 2 Faculty of Environment, Harbin Institute of Technology, Harbin, 15001 China

**Keywords:** Biological techniques, Environmental sciences, Energy science and technology, Engineering

## Abstract

To address issues of high water content and low calorific value during combustion of municipal sludge, we added water-absorbent, easy-to-burn agricultural waste to improve the overall combustion performance. Cotton straw or corn stover were added to the sludge and mixed at high-speed to compare their capacities for improving combustion performance. Scanning Electron Microscopy (SEM) revealed that cotton straw or corn stover attached to the surface of the municipal sludge particles after blending, while analysis of thermogravimetric curves and activation energies of the blends showed that combustion and exhaustion rates increased significantly when 40% cotton straw or corn stover were blended into the sludge. Using the quadrilateral cut-ring boiler as a prototype, the mix of sludge with cotton straw or corn stover was simulated, and FLUENT software was used to obtain the temperature and pollutant emissions of the boiler. Sludge blended with cotton straw or corn stover increased furnace temperature and reduced SO_2_ and NO emissions, while that with cotton straw burned at higher temperatures with lower SO_2_ and NO emissions. Overall, the CO content of sludge combustion was lower when blended with proportions of cotton straw or corn stover under 50%. The findings of this study lay a theoretical foundation for treatment of municipal sludge according to local conditions.

## Introduction

Various industries have developed carbon reduction methods as part of the “dual carbon” goals in China. In the western region, thermal power plants are prioritizing energy conservation and environmental protection by achieving carbon and consumption reduction. Additionally, alternatives to traditional coal fuel have become a popular research topic. Municipal sludge, regarded as solid waste, is considered a type of "fuel"^1^ due to its certain calorific value and required processing amount^2^. The "Urban Domestic Wastewater Treatment Facilities to Make Up for the Shortfall" covers waste treatment; specifically, the "Implementation Program of Mending Shortcomings and Strengthening Weaknesses of Urban Living Sewage Treatment Facilities"^3^ states that "large and medium-sized cities in the central and western regions of China must reduce the scale of sludge landfills. In large and medium-sized cities where land resources are scarce, the use of 'biomass utilization + incineration' disposal mode is encouraged".

Xinjiang is located in the northwestern border of China; thus, it acts as an important gateway connecting Central Asia and Europe and as an important node of the "One Belt, One Road" strategy. Its geographic location provides Xinjiang with a wide space and opportunities for development and cooperation; thus, Xinjiang is one of China's major agricultural provinces and the largest producer of cotton^4^. However, agricultural waste in this region has a low utilisation rate^5,6^, necessitating investigation of the performance characteristics of waste for use in synergistic resource utilisation, sustainable waste treatment, and the establishment of a low-carbon, energy-efficient, and environmentally friendly municipal sludge processing strategy.

Researchers have attempted to co-combust municipal sludge and biomass to improve sludge combustion. For example, Yuanyuan et al^7^. (2022) reported that when 60% sludge was blended with 40% peanut shells, the combined combustion performance index increased by 6.9 times, the volatile release characteristic index increased by 4.5 times, and the activation energy decreased by 19.43 kJ/mol. Additionally, Geon-Uk Baek^8^ et al. (2020) reported that reduction of SO_2_, NO and CO by 70.8, 77.1 and 38.2%, respectively, for 50%t sludge versus 50% woody biomass combustion. Wang^9^ et al. (2020)investigated the combustion and emission characteristics of sludge mixed with biomass, such as rice husks, and concluded that the NO_x_ and SO_2_ emissions from 80% biomass + 20% sludge were lower than those from sludge combustion, NO_x_ was reduced by about 66% and SO_2_ by 80%. Further, Jingyong Liu^10^ et al. (2018) concluded that 60.1% blending of sewage sludge and water hyacinth resulted in the best combustion parameters at a heating rate of 29.9˚C/min, and a maximum burnout rate of 92.4%. Jin-Ho Sung^11^ et al. (2018)concluded that 70%sludge + 30% biomass, with 23% oxygen mixing rate When burning in a 30 kWth CFB, lowest temperature gap between riser and stack occurred, high enrichment of CO_2_ (over 90%) with less pollutant emissions, specifically 0.91% CO and 14 ppm NO.

**Figure 1:**
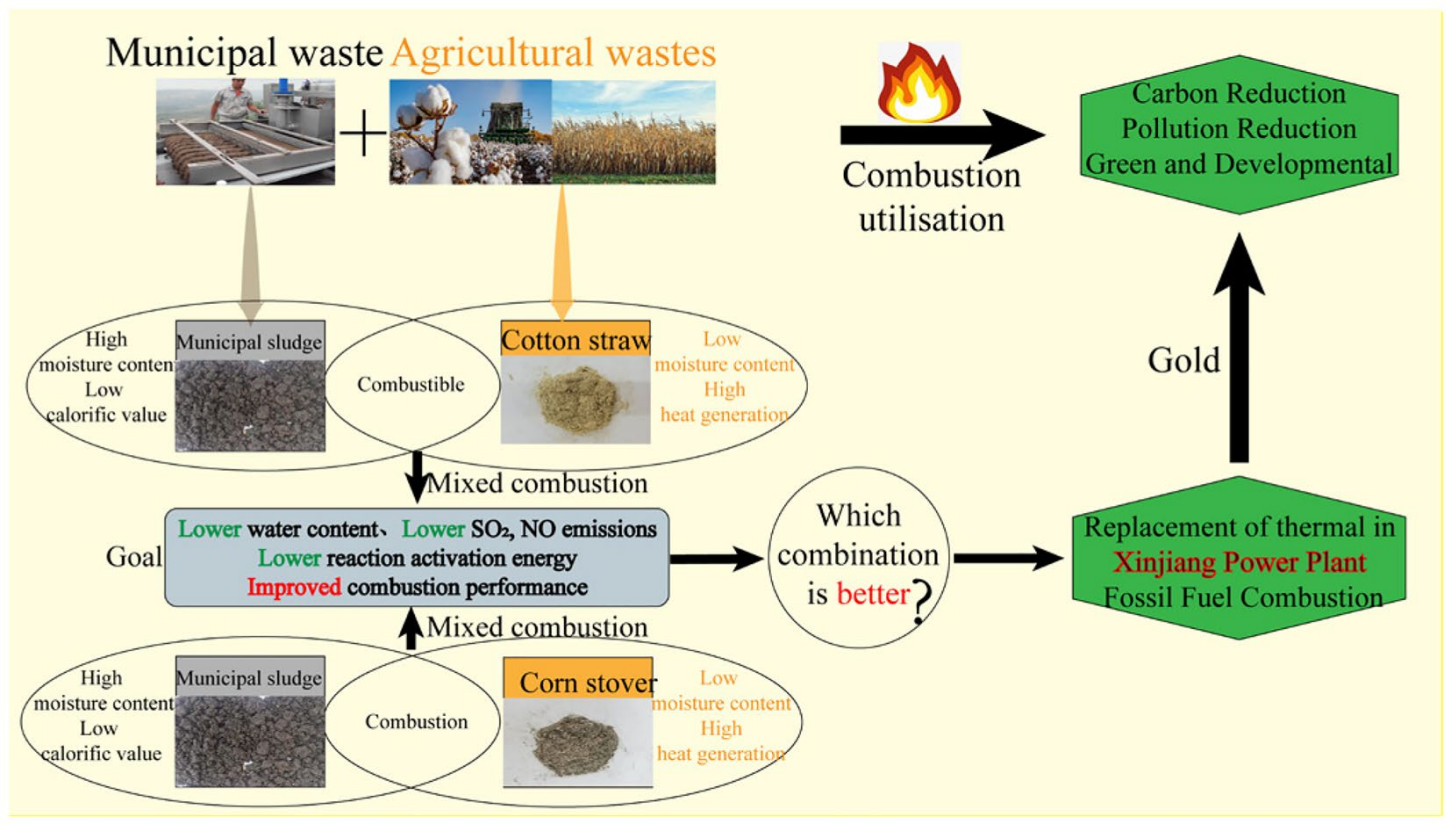
Conceptualization of municipal Sludge Combustion in Xinjiang.

Given the large-scale production of urban sludge containing high moisture^12^ content, low calorific value^13,14^, low level of resource utilization and low recycling of energyas, as well as that of cotton straw and corn stover (containing high cellulose and hemicellulose contents and calorific value)^15–17^, in Xinjiang, this study features a comparative analysis on the combustion performance of urban sludge after addition of different proportions of cotton straw or corn stover waste. Using the advantages of Computational Fluid Dynamics (CFD), such as high operability, low cost, and effective observation of the combustion process^18^, the temperature and flue gas distribution of sludge mixed with cotton straw (or corn stover) in a quadrangular cut-circle boiler were solved by Fluent software. Studies of this nature are critical in the search for new biomass fuels to replace fossil fuels in thermal power plants, and for providing theoretical support for cleaner production while synergistically promoting carbon and pollution reduction. A schematic diagram of the synergistic resource utilisation strategy for treatment of municipal sludge is shown in Fig. 1.

## Materials and methods

### Materials and pre-processing

The experimental sludge was obtained from the domestic wastewater treatment plant (WWTP) ex-factory sludge with 80% water content. The wastewater treatment process of this wastewater treatment plant is activated sludge method. The effluent sludge is the sludge after dosing, disinfection and mechanical dewatering and is disinfected. The experimental mud is not dried above 60 °C. The sludge is a zoogloea and there is no bacteria treatment during the whole process. The cotton straw used in the experiment were obtained from cotton straw of long-staple cotton harvested in autumn in Xinjiang. The corn stover used in the experiment came from the corn stover of MC670 corn after harvesting in autumn in Xinjiang. Natural air drying of sludge taken from sewage treatment plant, and then subject to the use of RRH-100 crusher, take 0.075 mm sludge particles for spare.Cotton straw, and corn stover were pre-processed the same way, the same RRH-100 crusher was used to crush and 0.075 mm particles were taken for spare.

### Experimental methods

#### Mixing methods

**Figure 2:**
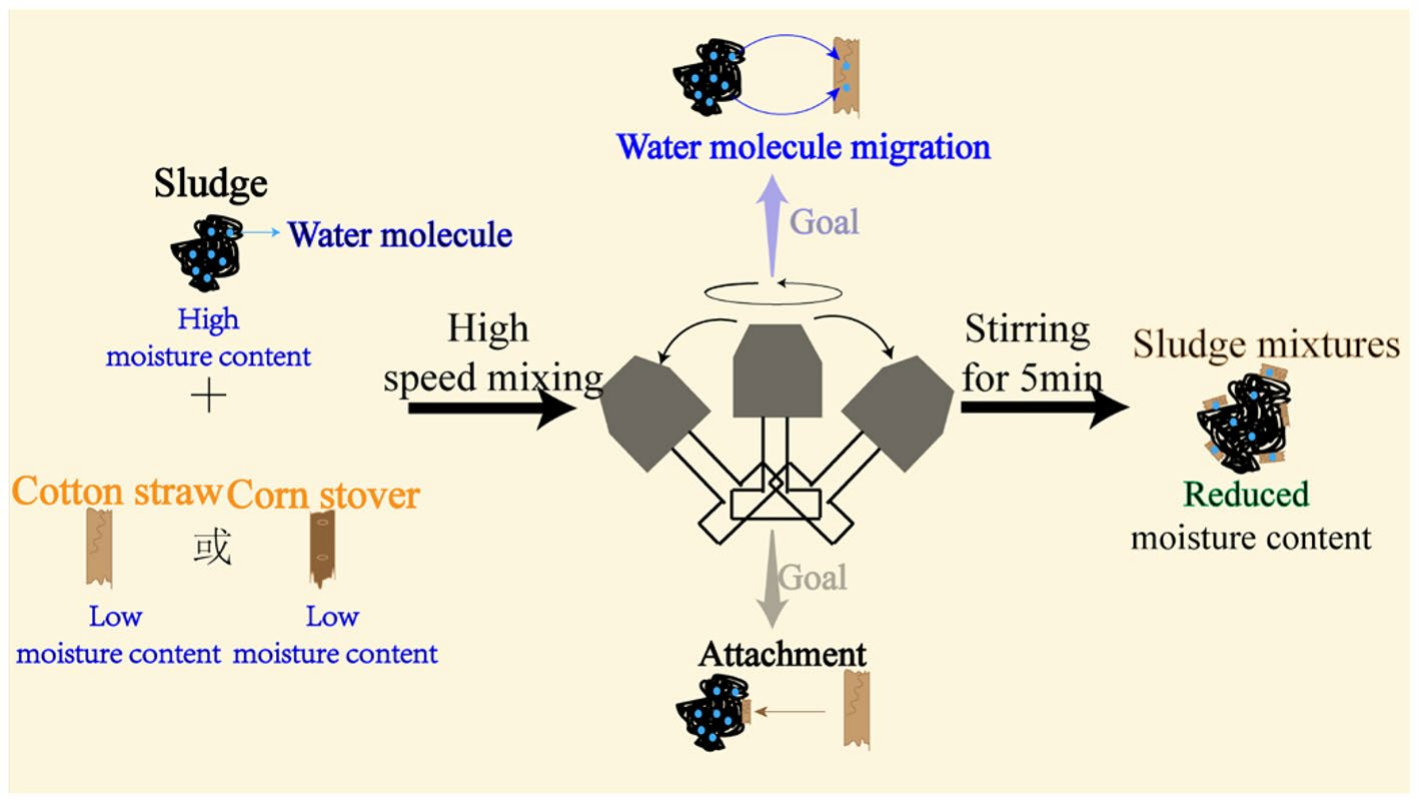
Schematic diagram of the method for blending sludge with cotton straw or corn stover.

An RRH-100 crusher, set at a mixing speed of 28,000 rpm, was used as the mixing device. Figure 2 shows a schematic diagram of the blending process. Sludge with a particle size of 0.075 mm was placed in a mixing unit with cotton straw (or corn stover) and stirred for 5 min. The mixing device oscillated at a uniform speed during the mixing process, which promoted the homogeneous mixing of the sludge with the cotton straw or corn stover.

#### Elemental and industrial analyses and calorific value

The elemental contents of C, H, O, and N, as well as moisture, ash, volatile matter, fixed carbon content, and calorific value of the sludge, cotton straw, and corn stover were measured in accordance with The Elemental Analysis of Coal (GBT31391-2015), The Industrial Analysis of Coal (GBT212-2008), and Determination of the Coal Heat Generation Method (GBT213-2008). Table 1 shows the instrument types and models.

**Table 1:** Instrument type and model.

Name	Type	Place of origin
SHCL-800	Sulfur analyzer	China
SDCHN536	CHN analyzer	China
HYHW-8A	Automatic calorimeter	China
TQ-3	CH analyzer	China

#### Microstructure analysis

Scanning electron microscopy was carried out using a German Zeiss SEM field emission scanning electron microscope GeminiSEM 300 for microstructure observation. A 10 mg substrate, with a particle size of 0.075 mm, was glued to the conductive adhesive, then 45 s gold spraying with 10 mA using a Quorum SC7620 sputter coater. Working distance is 8.5 mm. The acceleration voltage was set to 3 kV and a magnification of 2000X for morphology filming. The microstructures of sludge, cotton straw, and corn stover were observed and analysed before and after blending.

#### Thermogravimetric analysis

Thermogravimetric experiments were performed using a German NETZSCH TG209F1 analyser. The heating rate was 20 °C/min, starting at 25 °C and reaching 1000 °C. Throughout the experiment, the atmosphere was ambient air, with an air intake rate of 60 ml/min. The thermodynamic curves of the sludge, cotton straw, and corn stover were analysed in individually and in combinations of sludge with corn stover or cotton straw^19,20^.Table 2 shows the technical parameters of thermogravimetric analyzer.

**Table 2:** Thermogravimetric analyzer (TG/DTG) technical parameters.

TG209F1	
Type	TG209F1
Brand	NETZSCH
Place of origin	Germany
Measuring range	10–1100℃
Rate of heating and rate of cooling	0.001–200 K/min
Weighing range	2000 mg
Atmosphere	Inert, oxidizing, reducing, static, dynamic

#### Kinetic analysis

Activation energy is the energy required for a substance to change from its normal state to an active state; the higher the activation energy, the more difficult it is for a substance to react chemically^21^. In this experiment, the Coats-Redfern^22,23^ model was used to calculate the activation energy of an inhomogeneous system under non-isothermal conditions, using Eqs. 1 and 2:

1 $$\frac{{{\text{d}}\alpha }}{{{\text{dT}}}} = \frac{A}{\beta }\exp \left( { - \frac{E}{RT}} \right)f\left( \alpha \right)$$

2 $$\alpha = \frac{{m_{0} - m_{t} }}{{m_{0} - m_{\infty } }}$$

where A is the prime factor; $\beta$ is the rate of temperature increase, °C/min; E is the activation energy, kJ/mol; R is the ideal gas constant, 8.314 J/mol-K;$T$ is the thermodynamic temperature, K;$\alpha$ is the percent conversion; ${m}_{0}$ is the initial mass of the sample, mg; ${m}_{\infty }$ is the sample reaction termination mass, mg; ${m}_{t}$ is the sample mass at time t, mg; $f(\alpha )$ is the mechanism function; and $G\left(\alpha \right)$ is the kinetic model function.

Through integration of Eq. (1), we get Eq. (3),

3 $$\int_{0}^{\alpha } {\frac{d\alpha }{{f\left( \alpha \right)}}} = \frac{A}{\beta }\int_{{T_{0} }}^{T} {\exp \left( { - \frac{E}{RT}} \right)} dT$$

where $G\left(\alpha \right)={\int }_{0}^{\alpha }\frac{{\text{d}}\alpha }{f(\alpha )}$.Using the Coats-Redfern model, the integral of Eq. (3) takes the logarithmic form, as expressed in Eq. (4):

4 $$\ln \left[ {\frac{G\left( \alpha \right)}{{T^{2} }}} \right] = \ln \left( {\frac{AR}{{\beta E}}} \right) - \frac{E}{RT}$$

where *1/T* represents the horizontal axis and ln(G($\alpha$)/2 ) represents the vertical axis. The function was linearly fit to find the activation energy, and pre-exponential factor A.

#### Simulation analysis

In this study, a 330 MW quadrangular tangent circle commonly used in Xinjiang is used for numerical simulation. The diameter, width, and height of the boiler were 12.80 m, 12.80 m, and 55.23 m, respectively. The operating parameters of the boiler are listed in Table 3 and the simulated combustion feed ratios are listed in Table 4.

**Table 3:** Operational parameters of the 330MW quad-cut circle combustion furnace setup.

Name	Primary wind speed (m/s)	Primary air temperature (℃)	Secondary wind speed (m/s)	Secondary air temperature (℃)	Burn-out wind speed (m/s)	Burnout wind temperature (℃)	Inlet temperature (℃)	Mean static pressure (Pa)	Wall temperature (℃)	Grain size (mm)
Value	27	69.85	46	360.85	46	360.85	69.85	− 80	926.85	0.075~0.6

**Table 4:** Simulated combustion feed ratios of sludge, cotton straw, and corn stover for a 330 MW quad-circular combustion furnace.

working condition	Sludge (%)	Cotton straw (%)	Corn stover (%)
1	100	0	0
2	90	10	0
3	80	20	0
4	70	30	0
5	60	40	0
6	50	50	0
7	90	0	10
8	80	0	20
9	70	0	30
10	60	0	40
11	50	0	50

**Figure 3:**
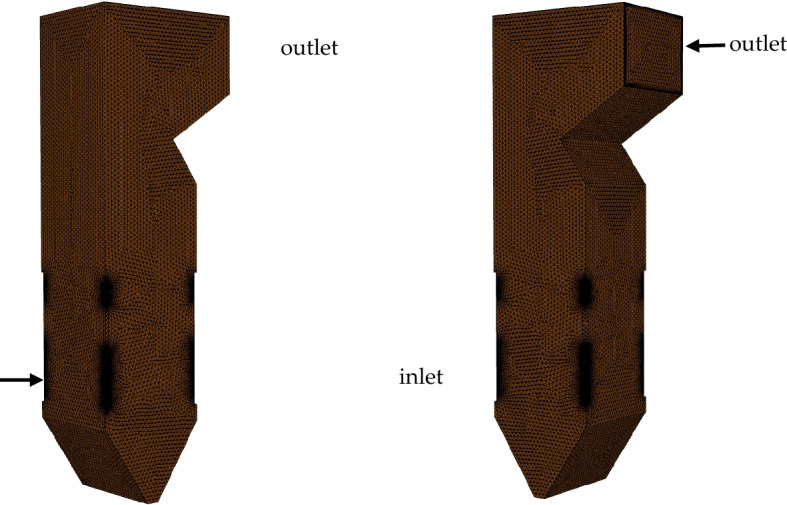
Modelling and meshing of a four-corner, cut-circle combustion furnace.

In this study, Fluent 14.0 from Ansys software was used, which consists of 565,206 structural units, as shown in Fig. 3. The grid was refined at important locations, such as primary air nozzles and material nozzles. The modelling and computational methods considered for the validity and accuracy of the model were as follows:

1. Standard modelling equations including the conservation of mass, conservation of energy, conservation of momentum, and conservation of substance concentration equations were used^24^.
2. A matter transport model was combined with a discrete-phase model to simulate boiler combustion^25^.
3. A simulation of turbulent gas flow in pulverised coal boilers was carried out using the standard j-e turbulence model^26^.
4. Radiative heat transfer was calculated using the P-1 radiation model, because heat transfer affects combustion and particle behaviour^27^.
5. The surface reaction of the fuel combustion was modelled using a finite rate/vortex dissipation model^28^.
6. The NO_X_ model primarily considered thermal NO_X_ and fuel NO_X_^29,30^.

## Results and discussion

### Elemental analysis and industrial analysis

The experiments were carried out according to the experimental methods in “Elemental and industrial analyses and calorific values” and the results are shown in Table 5. The C and H contents of the sludge were lower than those of the cotton straw or corn stover, while the contents of O, N, and S were higher than those of the cotton straw or corn stover. Generally, greater amounts of C correlate with greater heat generation, while high H content makes ignition and burning easier. However, higher O contents can inhibit combustion^31^. Therefore, cotton straw or corn stover has a higher heat generation during combustion than sludge, and ignition and combustion are easier than sludge. The industrial analysis showed that the sludge moisture and ash contents were higher than those of cotton straw and corn stover, but the volatile fraction and fixed carbon ratios were lower. Lower moisture and ash contents reduce energy consumption^32,33^, thereby improving the thermal efficiency of combustion; meanwhile, higher volatile matter and fixed carbon ratios, generate more heat during combustion, improving the utilisation rate of combustion. This result suggests that cotton straw and corn stover require less energy consumption during combustion and maintain higher thermal efficiency and heat generation than does sludge alone.

**Table 5:** Elemental analyses, industrial analyses, and calorific values of sludge, cotton straw, and corn stover.

Sample	Elemental analysis (%)					Industrial analysis (%)				${Q}_{net,d}$ (MJ/kg)
	${C}_{daf}$	${H}_{daf}$	${O}_{daf}$	${N}_{daf}$	${S}_{daf}$	${M}_{ad}$	${A}_{ad}$	${V}_{ad}$	${FC}_{ad}$	
Sludge	32.84	3.86	57.53	5.06	0.71	19.68	30.52	43.34	6.46	11.05
Cotton straw	48.70	6.05	44.67	0.47	0.11	5.86	5.12	70.21	18.81	16.94
Corn stover	50.26	6.20	42.36	1.00	0.18	6.62	6.02	67.04	20.32	16.80

### Microstructure analysis

**Figure 4:**
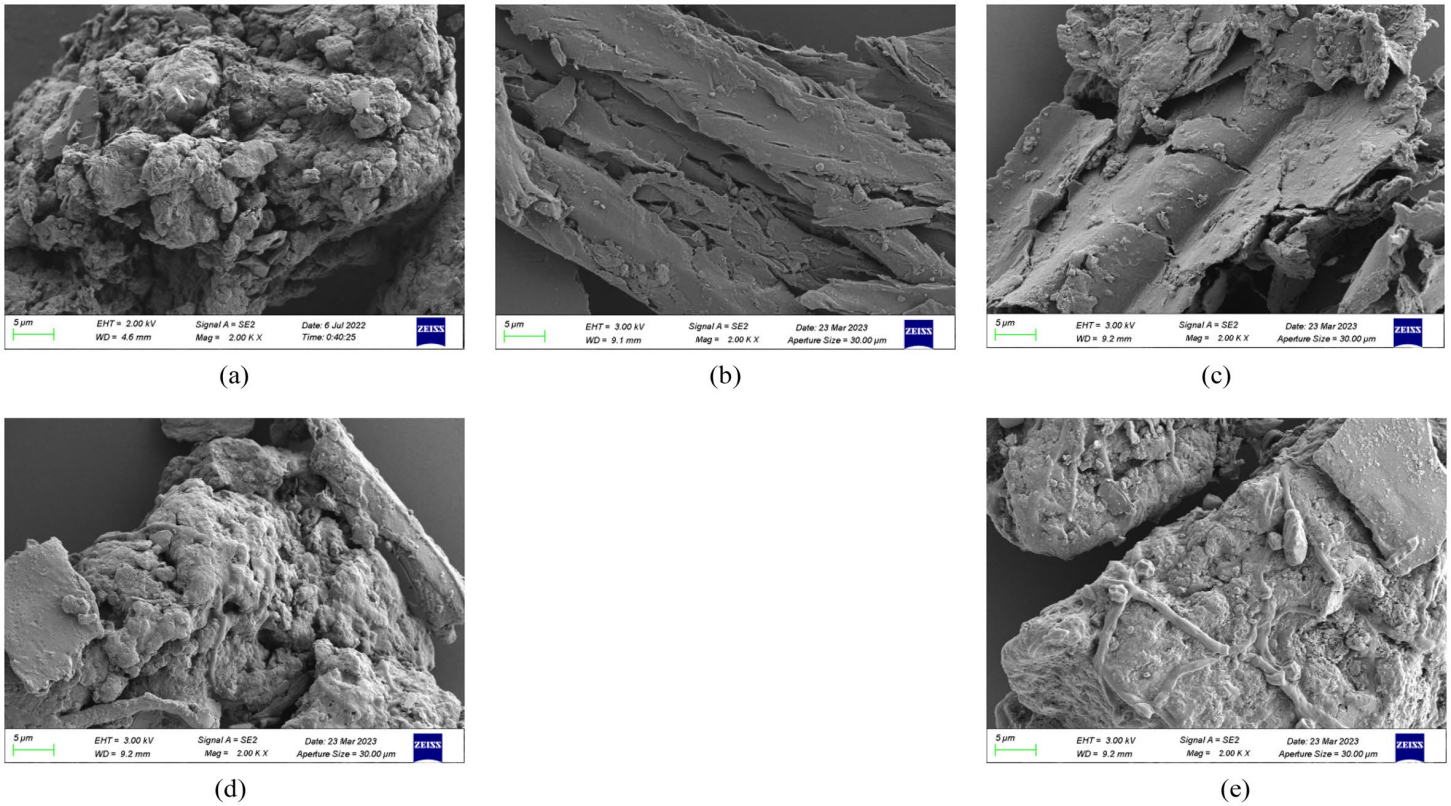
Scanning electron micrographs of the microstructures of sludge, cotton straw, and corn stover before and after blending. (**a**) Cotton straw; (**b**) Sludge; (**c**) Corn stover; (**d**) Sludge + cotton straw; (**e**) Sludge + corn stover.

The SEM images of the sludge, cotton straw, and corn stover before and after blending are shown in Fig. 4. As shown in Fig. 4a, the sludge consisted of irregularly sized particles with rough surfaces, complex lumpy structures, and pores of different sizes. As shown in Fig. 4b, cotton straw had a laminar void and a relatively loose structure. As shown in Fig. 4c, corn stover also had a lamellar structure, but with a denser outer surface. As shown in Fig. 4d,e, cotton straw and corn stover particles were attached to the surface of the sludge particles after blending.

### Thermogravimetric analysis

**Figure 5:**
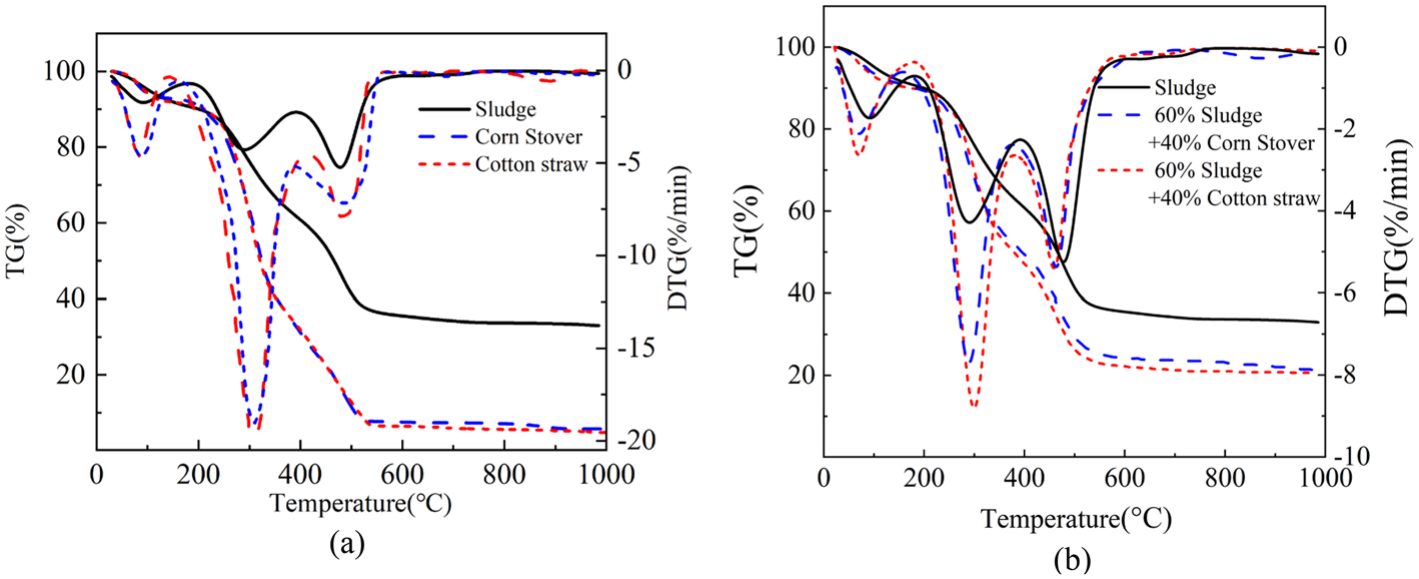
Thermogravimetric curves. (**a**) Thermogravimetric curves of sludge, cotton straw, and corn stover burned individually; (**b**) thermogravimetric curve of combustion of sludge blended with cotton straw and corn stover.

Relevant data show that when sludge and other substances are mixed and burned, the water content is reduced to 30–40%^34^, which is conducive to increasing the temperature of combustion, and the combustion is more stable^25^. So this study selected 60% sludge and 40% cotton straw for mixing combustion, thermogravimetric experiment results are shown in Fig. 5a. The weight loss stages of the sludge were the same as those of cotton straw and corn stover across all three stages^35^; however, the maximum rate of weight loss and final weight loss rate in the three stages of cotton straw and corn stover was greater than that of the sludge, indicating that the combustion of cotton straw and corn stover was more complete, and the burning rate was higher than that of sludge alone^36^.

Figure 5b shows the thermogravimetric curves of the sludges mixed with cotton straw or corn stover. The TG-DTG of both sets of mixed samples showed three phases, each of which occurred at approximately the same temperature interval as in the case of sludge burning alone, indicating that mixing sludge burning with these two types of agricultural waste did not alter the weight loss phase. The blended combustion increased the weight loss rate across the three stages, but the weight loss rate of the 60% sludge + 40% cotton straw was greater^37^ than that of the 60% sludge + 40% corn stover ratio, and the blended combustion left less residue, suggesting that the addition of the agricultural waste effectively improved the sludge combustion rate, made the sludge burn more fully, and resulted in a higher burnout rate^38^.

### Non-isothermal kinetic fitting and activation energy study

**Figure 6:**
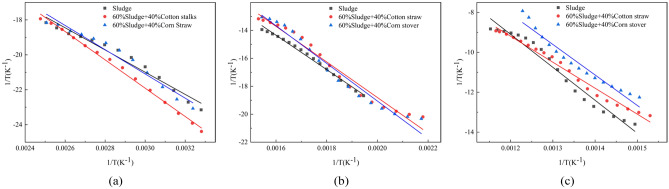
Kinetic fitting of sludge, cotton straw and corn stover combustion alone and blended combustion. (**a**) The first stage; (**b**) The second stage; (**c**) The third stage.

The results of non-isothermal kinetic fitting are shown in Fig. 6 and the results of the activation energy calculations are shown in Table 6. The average activation energy of sludge alone was 116.65 kJ/mol, and the addition of cotton straw or corn stover reduced the activation energy of sludge by 12.74 kJ/mol for the 60% sludge + 40% cotton straw mixture and 5.58 kJ/mol for the 60% sludge + 40% corn stover mixture. Therefore, the addition of cotton straw or corn stover promoted sludge combustion^39^, though the cotton straw mix had a stronger positive effect. Taken together, these results suggest that the mixture of 60% sludge + 40% cotton straw produced the lowest reaction activation energy of sludge combustion, and thus exhibited better combustion performance^40,41^.

**Table 6:** Elemental analyses, industrial analyses, and calorific values of sludge, cotton straw, and corn stover.

Materialistic	Stage	Activation energy ${\varvec{E}}$ (kJ/mol)	Average activation energy ${{\varvec{E}}}_{{\varvec{m}}}$ (kJ/mol)	Indexing factor ${\varvec{A}}$ (min^−1^)	Percentage weight loss ${\varvec{f}}$ (%)
Sludge	1	67.36	116.65	1.79 × 10^3^	9.53
	2	103.14		6.23 × 10^4^	24.19
	3	141.30		2.89 × 10^7^	32.31
60% Sludge + 40% Cotton straw	1	52.85	103.91	1.86 × 10	8.83
	2	109.55		4.55 × 10^5^	49.27
	3	110.98		2.56 × 10^5^	24.44
60% Sludge + 40% Corn Stover	1	57.68	111.07	1.58 × 10^2^	8.89
	2	111.93		1.06 × 10^6^	45.44
	3	127.13		1.33 × 10^7^	27.11

### Numerical simulation of boiler combustion

#### Temperature cloud of sludge mixed with cotton straw combustion

**Figure 7:**
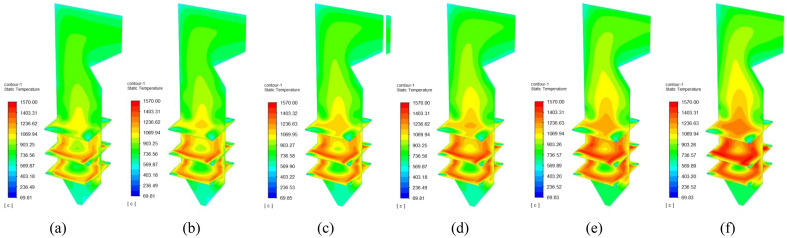
Temperature cloud of sludge and cotton straw combustion at different blending ratios. (**a**) Sludge; (**b**) 90% sludge + 10% cotton straw; (**c**) 80% sludge + 20% cotton straw; (**d**) 70% sludge + 30% cotton straw; (**e**) 60% sludge + 40% cotton straw; (**f**) 60% sludge + 40% cotton straw.

The variations in boiler temperature for the combustion of sludge and cotton straw at different blending ratios are shown in Fig. 7. Temperature cloud of sludge and cotton straw combustion at different blending ratios. (a) Sludge; (b) 90% sludge + 10% cotton straw; (c) 80% sludge + 20% cotton straw; (d) 70% sludge + 30% cotton straw; (e) 60% sludge + 40% cotton straw; (f) 60% sludge + 40% cotton straw The six groups of hearth temperature cloud maps were ultimately similar in distribution, and the temperature fields were all relatively stable, indicating that the addition of cotton straw did not affect the flame combustion pattern in the hearth. With an increase in the cotton straw blending ratio, however, the furnace chamber temperature gradually increased. The highest temperature of sludge combustion alone was 1485.60℃, while the maximum temperature was 1564.12 °C after adding 50% cotton straw, likely because the addition of cotton straw increased the fixed carbon content and reduced the moisture and ash contents of the sludge, resulting in a higher combustion temperature in the furnace.

#### Temperature cloud of sludge mixed with corn stover combustion

**Figure 8:**
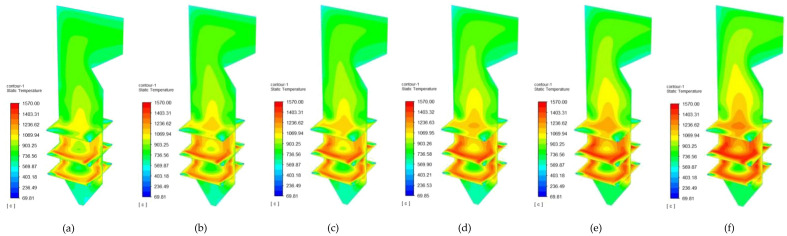
Temperature cloud of sludge and corn stover combustion at different blending ratios. (**a**) Sludge; (**b**) 90% Sludge + 10% Corn stover; (**c**) 80% sludge + 20% corn stover; (**d**) 70% sludge + 30% corn stover; (**e**) 60% sludge + 40% corn stove; (**f**) 50% sludge + 50% corn stover.

The boiler temperature variations of the sludge and corn stover combustion at different blending ratios are shown in Fig. 8.

The simulation results for the combustion of the sludge and corn stover blends appear similar to those for the sludge and cotton straw blends, with the furnace chamber temperature increasing with the increase in corn stover content. When the proportion of corn stover was 50% sludge to 50% corn stover, the highest temperature, 1547.92℃, was reached, likely because the addition of corn stover reduced the moisture and ash contents of the sludge and increased the fixed carbon content, thereby increasing the combustion temperature of the furnace.

#### Pollutant emissions from co-combustion of sludge and cotton straw

**Figure 9:**
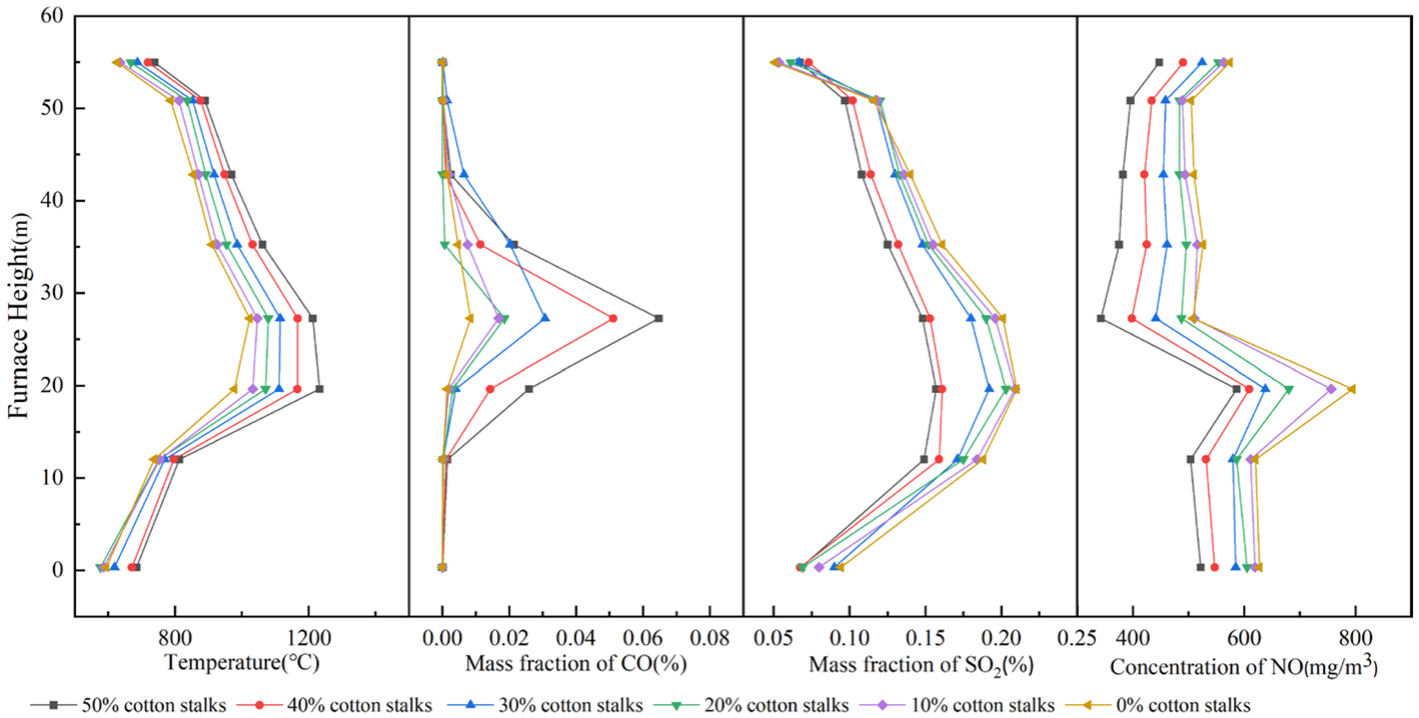
Temperature and pollutant emission distribution of sludge and cotton straw combustion at different blending ratios.

Figure 9 shows the temperature and pollutant emission profiles of sludge and cotton straw combustion under different blending ratios. Based on the temperature curves, the overall trend of the six sets of curves was similar, with the temperature first increasing and then slowly decreasing as the height of the hearth increases^42^. The high-temperature region of furnace combustion occurred in the main combustion region, which is consistent with actual boiler operation^43^. As the proportion of cotton straw increased, the combustion temperature in the furnace chamber also gradually increased.

The CO mass fraction curve with hearth height first increased and then decreased. CO mass fraction increased with the increase of cotton straw blending ratio, the sludge combustion alone for 8.42 × 10^–5^, blended with 50% cotton straw for 6.47 × 10^–4^, indicating that greater levels of cotton straw during combustion increased the production of CO. This is because the temperature of the chamber increases more rapidly for a larger proportion of cotton straw; thus, the mixture burns faster and the surrounding oxygen is consumed at a faster rate than the incoming oxygen, resulting in insufficient local burning and an increase in the mass fraction of CO^44^.

SO_2_ production appeared to increase and then decrease with the increase in furnace height; in the main combustion zone, the sludge combustion of the SO_2_ mass fraction was the largest, 2.10 × 10^–3^. The SO_2_ mass fraction peaks reduced significantly with a larger fraction of cotton straw; specifically, blending with 50% cotton straw resulted in the lowest production of SO_2_. The formation of SO_2_ is closely related to the sulfur content of the material. The content of elemental S in sludge was higher than that of cotton straw, therefore, with an increase in the cotton straw blending ratio, the content of elemental S decreased, and the emission of SO_2_ decreased.

The NO concentration increased and then decreased along the hearth height, with a slight increase near the flue gas outlet. The NO concentration was mainly concentrated near the main combustion zone, which had the highest temperature and produced large amounts of fuel- and thermal-type NO_x_^45^. Lower temperatures in the lower and upper parts of the boiler reduce thermal NO_x_ production. There was a slight increase in the NO concentration near the furnace exit, likely due to the increase in NO emissions resulting from poorer combustion performance. As the percentage of cotton straw increased, the concentration of NO decreased gradually, while the highest value of NO concentration for sludge combustion alone was 794.42 mg/m^3^, and 586.41 mg/m^3^ after blending with 50% cotton straw, which resulted in a decrease of 208.01 mg/m^3^.

#### Pollutant emissions from combustion of sludge with corn stover

**Figure 10:**
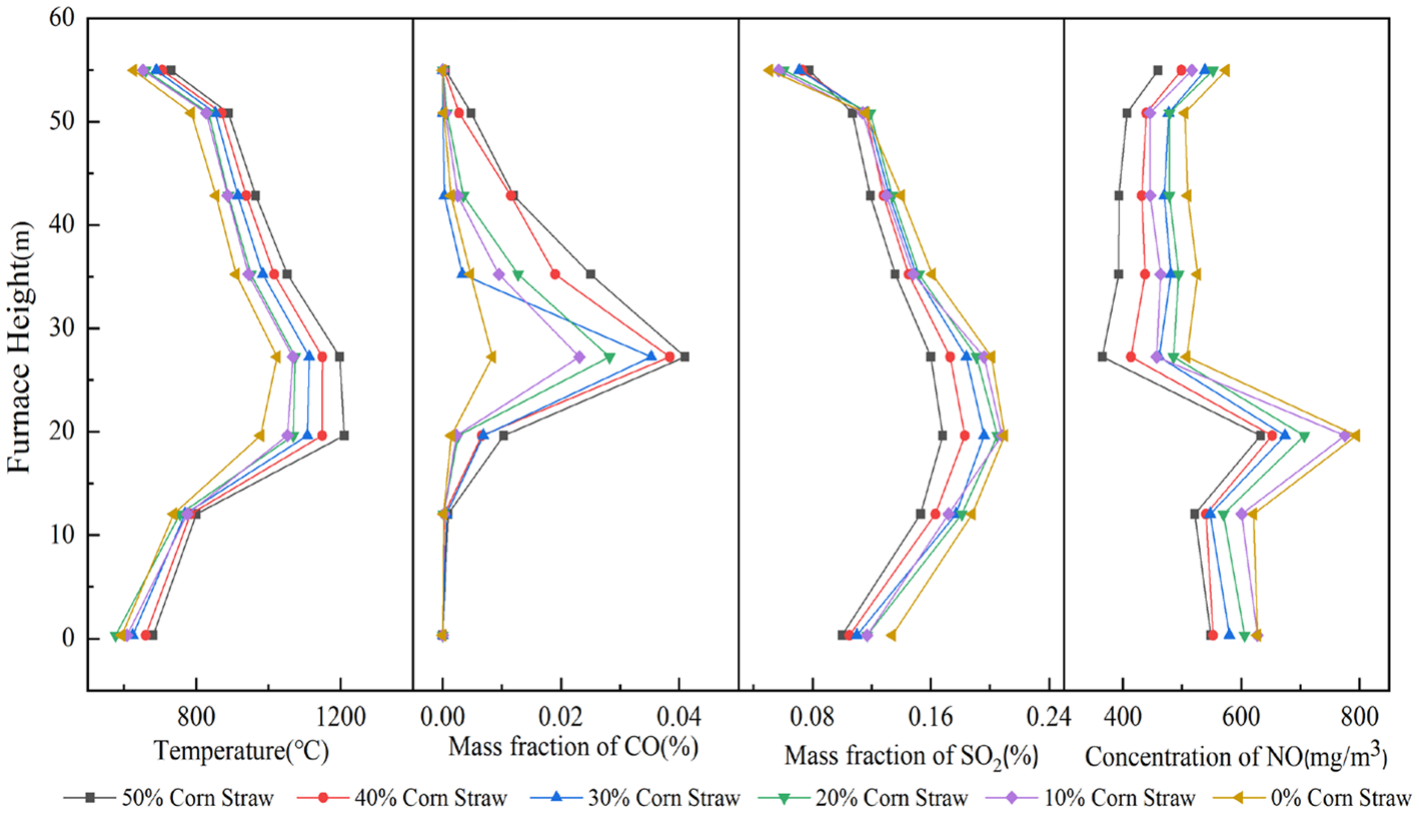
Temperature and pollutant emission distribution of sludge and corn stover combustion at different blending ratios.

Figure 10 shows the temperature and pollutant emission profiles of sludge and corn stover combustion for different blending ratios. With an increase in the height of the furnace chamber, the temperature first rose and then slowly decreased, and the temperature inside the furnace chamber gradually increased as the proportion of corn stover increased in the blend.

The CO mass-fraction profile peaked in the main combustion zone and approached zero near the slag and smoke outlets. The maximum CO mass fraction was 4.1 × 10^–4^ for the with 50% corn stover blend. The higher the corn stover proportion in the blend, the higher the resulting CO mass fraction.

The SO_2_ trend was similar to that of the boiler combustion temperature, with high SO_2_ content in the high-temperature zone and low SO_2_ content in the lower part of the furnace and exhaust port. As the proportion of corn stover increased, the SO_2_ in the furnace chamber gradually decreased. The mass fraction of SO_2_ was 1.68 × 10^–3^ when mixed with 50% corn stover.

The NO concentration curve first increased then decreased, with the curve flattening at approximately 30 ~ 50 m furnace height followed by a slight increase at the flue gas outlet. The lowest NO concentration, 632.61 mg/m^3^, was produced from the 50% corn stover blend, and the NO concentration at the furnace exit also decreased with increasing corn stover blending ratio. This is consistent with the findings of Hongpeng Liu^46^ et al. (2021) that the incorporation of corn stover reduces NO and SO_2_ emissions.

#### Comparative analysis of sludge blended with different ratios of cotton straw and corn stover for combustion

**Figure 11:**
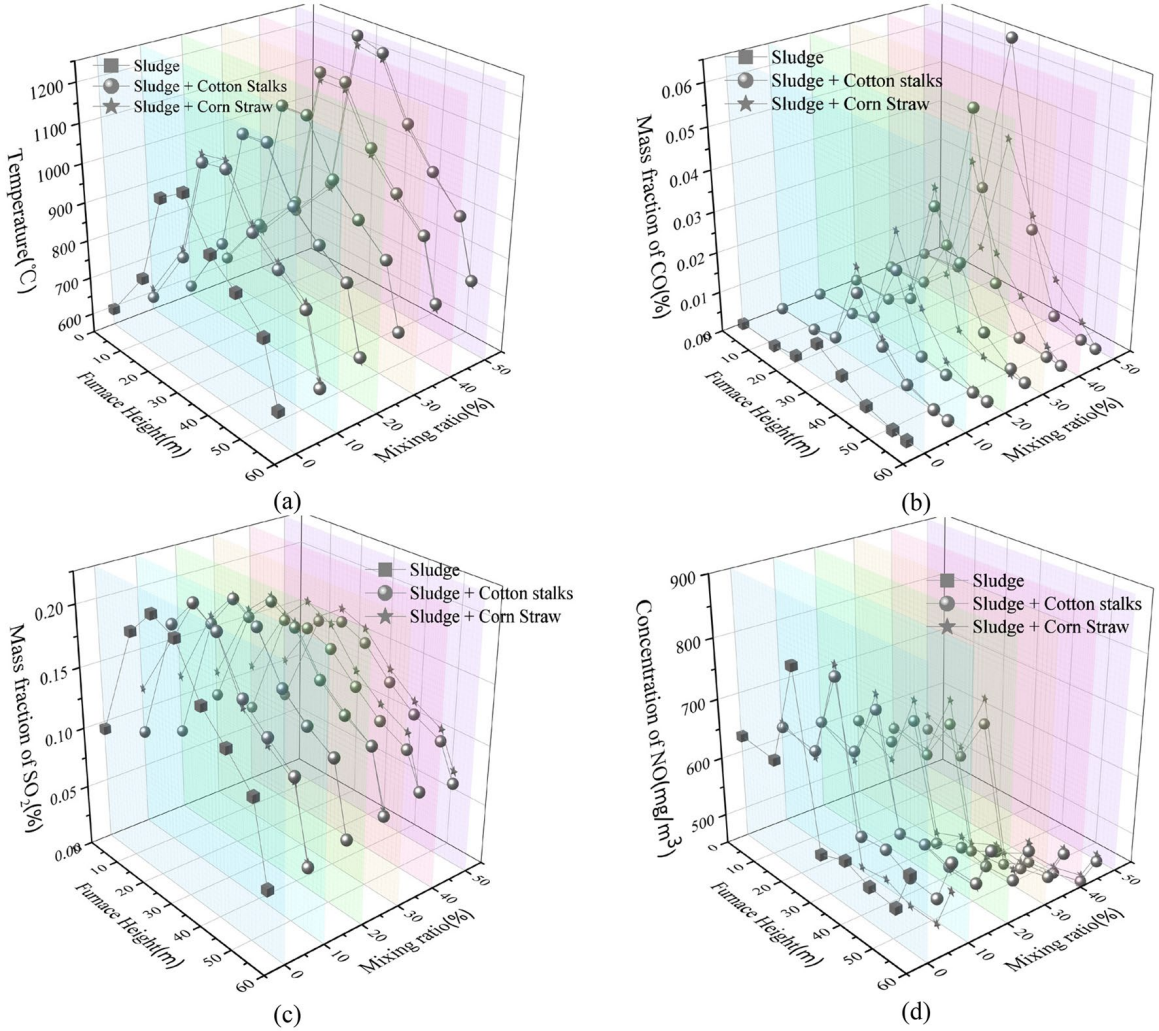
Comparison of combustion simulation results of sludge blended with cotton straw or corn stover. (**a**) Temperature; (**b**) CO mass fractions; (**c**) of mass fractions of SO2; (**d**) NO concentrations.

A comparison of the simulation results for the combustion of sludge blended with cotton straw or corn stover is shown in Fig. 11. Figure 11a shows that, with an increase in the proportion of cotton straw or corn stover, the combustion temperature in the furnace chamber gradually increased, with the greatest increase coming from the cotton straw mix. For the 50% cotton straw or corn stover sludge mixes, the temperatures increased by 226.24 °C and 173.65 °C, respectively.

The combustion of sludge mixed with either cotton straw or corn stover resulted in increased CO emissions. When the proportion of cotton straw or corn stover in the sludge blend was less than 30%, the amount of CO produced by the cotton straw blend was lower, whereas when the mixing amount of cotton straw or corn stover was 30%-50%, the amount of CO produced by the mixed combustion of sludge and corn stover was smaller.

Overall, the combustion of sludge mixed with cotton straw or corn stover reduced SO_2_ and NO emissions, which is due to the fact that SO_2_ and NO emissions are mainly determined by the sulphur and nitrogen content of the substance^47^. The elemental analysis shows that, under the same blending ratio, lower SO_2_ emissions from combustion of sludge mixed with cotton straw occurred due to lower elemental S in cotton straw. Moreover, NO is mainly composed of fuel and thermal types^48^, of which fuel types account for 60%–80% of all NO, and thermal types account for about 20%. Fuel-type NO content is mainly determined by the N-element content of the burning material. Mao Ya^49^ et al. (2017) found that the NO burned by cotton straw was lower than that of corn stover. Thus, given that cotton straw had only half the N content of corn stover^50^, the NO produced by blending cotton straw burning was lower than that of corn stover.

## Conclusions

1. Blending sludge with cotton straw or corn stover for combustion increased the rate of weight loss and combustion exhaustion and decreased the activation energy of the reaction. When a ratio of 60% of sludge to 40% of cotton straw was burned, the activation energy decreased by 12.74 kJ/mol, while that with corn stover decreased by 5.58 kJ/mol. In summary, the addition of cotton straw or corn stover improved combustion performance compared to that of sludge alone.
2. At the same blending ratio, the combustion temperatures of sludge and cotton straw were higher than those of sludge and corn stover. Specifically, the furnace temperature increased by 226.24 and 173.65℃ when the percentages of cotton straw and corn stover, respectively, were 50%.
3. The CO content in the boiler increased with increasing cotton straw or corn stover blending ratios. When the ratio of cotton straw or corn stover was less than 30%, the CO content of the sludge and cotton straw blending and combustion was smaller. When the ratio of cotton straw to corn stover was 30%-50%, the CO content of the sludge and corn stover blending and combustion was smaller.
4. The SO_2_ and NO contents in the boiler gradually decreased with an increase in the blending ratio of cotton straw or corn stover. Because the S and N contents of the cotton straw were lower than those of the corn stover, the SO_2_ and NO contents produced by the mixed burning of sludge and cotton straw were lower than those of sludge and corn stover at the same ratio.

Experimental studies and finite element simulations have shown that mixing sludge and cotton straw for combustion can be more efficient in reducing the activation energy required for combustion, increasing the temperature of blended combustion, and reducing the emission of pollutants from blended combustion, compared to blending with corn stover. Therefore, a more in-depth study on sludge and cotton-straw-blended combustion should be carried out to further optimise the blending method and combustion conditions to improve the performance of sludge combustion in accordance with the Dual Carbon Strategy goals.

### Supplementary Information

Supplementary Information.

## Data Availability

The dataset used and analysed during the current study are available from the corresponding author (Jing Li) on reasonable request.
